# Discrimination of skyrmion chirality via spin–orbit and –transfer torques for logic operation

**DOI:** 10.1038/s41598-021-87742-6

**Published:** 2021-04-16

**Authors:** Yoshinobu Nakatani, Keisuke Yamada, Atsufumi Hirohata

**Affiliations:** 1 Graduate School of Informatics and Engineering, University of Electro-Communications, Chofu, Tokyo Japan; 2 Department of Chemistry and Biomolecular Science, Faculty of Engineering, Gifu University, Gifu-shi, Gifu Japan; 3 Department of Electronic Engineering, University of York, York, UK

**Keywords:** Magnetic devices, Magnetic properties and materials

## Abstract

Recently many works on magnetic memories and logic circuits, which use a magnetic skyrmion have been reported. Previously we micromagnetically simulated a method to switch a chirality of a magnetic skyrmion formed in a magnetic thin film by introducing a pulsed heat spot. In this paper, we propose a method to discriminate the chirality of a skyrmion in a branched nanowire by using spin–orbit torque (SOT) and spin-transfer torque (STT), and confirm the validity of the method by using simulation. The simulated results show that the motion changes depending on the chirality when additional SOT is applied on a skyrmion moving in a branch by STT. This method can be used as a fundamental building block for electrical detection in memory and logic devices using the chirality of skyrmions as a data bit in addition to the presence (and polarity) of the skyrmions as conventionally used, which can be lead to multiple-valued operation.

## Introduction

Magnetic skyrmion^1,2^ is a chiral structure appearing in a magnetic thin film by the Dzyaloshinskii-Moriya exchange interaction^3,4^. Because of the small size (~ 10 nm)^5,6^ and the small current density required for the motion (~ 10^6^ A/m^2^)^7–10^, a skyrmion is expected to become an ideal information carrier for next generation memory and logic devices such as a racetrack memory. Many works have accordingly been reported^9–22^. Among them, however, some works use the presence^9–12,14–19^ polarity^20^ or chirality^13,21^ of a skyrmion as a data bit, which are the other characteristics of the skyrmion^22^.

Previously we demonstrated by simulation that skyrmions with opposite chiralities can be formed in the same magnetic thin film, and their chiralities can be switched by introducing a single heat pulse^21^. These results show the possibility of the multiple-valued memory operation with skyrmions using their chirality. To date the chirality has been measured by imaging a skyrmion using magnetic force microscopy (MFM)^23,24^ and X-ray photoemission electron microscopy (XPEEM)^25^. However to realize such a memory, an electrical method to discriminate the chiralities is required.

Recently, it was reported that a skyrmion can be moved^26,27^ by using spin–orbit torque (SOT)^28–30^, and the direction of the motion changes depending on the chirality^31^. In this paper, we propose an electrical selection method to discriminate the chirality of the skyrmion by the combination of SOT and spin-transfer torque (STT)^32^, which is confirmed by simulation. When the SOT is applied on a skyrmion moving in a magnetic nanowire, the motion is found to be controlled depending on the chirality. By attaching a branch to the nanowire, the skyrmions with the opposite chiralities can be separated, achieving fully electrical detection of the chirality. This paves a way towards skyrmion memory and logic with multiple-valued operation using the skyrmions (and polarities) and their chiralities as data bits.

## Method

A micromagnetic model^33^ was used to calculate the motion of magnetic moments in a thin film using the Landau–Lifshitz-Gilbert equation with STT^32^ and SOT^30,34^ as follows:

1 $$\frac{\partial {\varvec{m}}}{\partial t}=-\left|\gamma \right|{\varvec{m}}\times {{\varvec{H}}}^{\mathrm{eff}}+\alpha {\varvec{m}}\times \frac{\partial {\varvec{m}}}{\partial t}-\left({\varvec{u}}\cdot \nabla \right){\varvec{m}}+\beta {\varvec{m}}\times \left[\left({\varvec{u}}\cdot \nabla \right){\varvec{m}}\right]-\frac{u{\theta }^{H}}{Ph}{\varvec{m}}\times \left({\varvec{m}}\times {{\varvec{n}}}_{{\varvec{s}}}\right).$$

Here, ***m***, ***H***^eff^, γ, and α are the unit magnetization vector that represents the direction of a local magnetic moment, the effective magnetic field acting on the magnetization, the gyromagnetic ratio, and the Gilbert damping constant, respectively. The velocity ***u*** is a vector along the direction of electron motion, with an amplitude of

2 $$u=jPg{\mu }_{\mathrm{B}}/\left(2e{M}_{\mathrm{s}}\right),$$

where *j, P, g*, *μ*_B_, *e*, and ${M}_{\mathrm{s}}$ are the current density, spin polarization, the Lande g-factor, the Bohr magneton, the electron charge, and the saturation magnetization, respectively. β is the dimensionless parameter for STT. ${\theta }^{H}$, *h*, and ***n***_s_ are the spin Hall angle, the thickness of the film and the unit vector of the spin Hall torque, respectively. The effective magnetic field is calculated from the magnetic energy density by ${{\varvec{H}}}^{\mathrm{eff}}=-\frac{1}{{M}_{\mathrm{s}}}\frac{\delta \varepsilon }{\delta {\varvec{m}}}$. For the magnetic energy, the exchange, anisotropy, demagnetizing, and Dzyaloshinskii-Moriya exchange interaction (DMI) energies are taken into account in the simulation^35,36^.

3 $$\varepsilon =A{\left(\nabla {\varvec{m}}\right)}^{2}+{K}_{u}\left(1-{m}_{z}^{2}\right)-\frac{1}{2}{M}_{s}{\varvec{m}}\cdot {{\varvec{H}}}^{dem}+D\left[\left({m}_{x}\frac{\partial {m}_{z}}{\partial x}-{m}_{z}\frac{\partial {m}_{x}}{\partial x}\right)+\left({m}_{y}\frac{\partial {m}_{z}}{\partial y}-{m}_{z}\frac{\partial {m}_{y}}{\partial y}\right)\right].$$

Here, *A*, *K*_u_, *H*^dem^, and *D* are the exchange stiffness constant, the uniaxial anisotropy constant, the demagnetizing field, which is calculated numerically, and the DM exchange constant, respectively. Here we assumed the interfacial DMI between the ferromagnetic thin film and the non-magnetic layer underneath. A free boundary condition was used at the edge of the film^36^.

4 $$\frac{d\theta }{dn}=\frac{1}{\xi } ,\quad \xi =\frac{2A}{D}.$$

Here, *θ* is a polar angle of a magnetic moment, *n* is the normal direction at the film edge, and *ξ* is a characteristic length determined by *D*^36^.

Typical material parameters for perpendicularly-magnetized CoFeB thin films at room temperature (300 K) were used, i.e., the saturation magnetization *M*_s_ = 1600 emu/cm^3^, the exchange stiffness constant *A* = 3.1 × 10^–6^ erg/cm, the uniaxial anisotropy constant *K*_u_ = 16.2 Merg/cm^3^, the gyromagnetic ratio γ = 1.76 × 10^7^ rad/(s Oe), the Gilbert damping constant *α* = 1, the dimensionless STT parameter β = 1, and the DMI constant *D* = 0.6 erg/cm^2^^37,38^.

In this paper, two types of simulation were performed. In the first one, we investigate the effect of the *D* value on the direction of the skyrmion motion by SOT. The second one is the simulation of the skyrmion motion in the nanowire with a branch by SOT and STT. In the first simulation, we use the magnetic strip with 512 nm in length, 128 nm in width and 1.4 nm in thickness. In the second simulation, the branch structure is made by attaching the same strips used in the first simulation. The branch angle is varied from 15 to 90 degree from the nanowire direction. These strips are divided into rectangular prisms with their dimensions of 0.5 × 0.5 × 1.4 nm^3^ in simulation.

## Results and discussion

**Figure 1:**
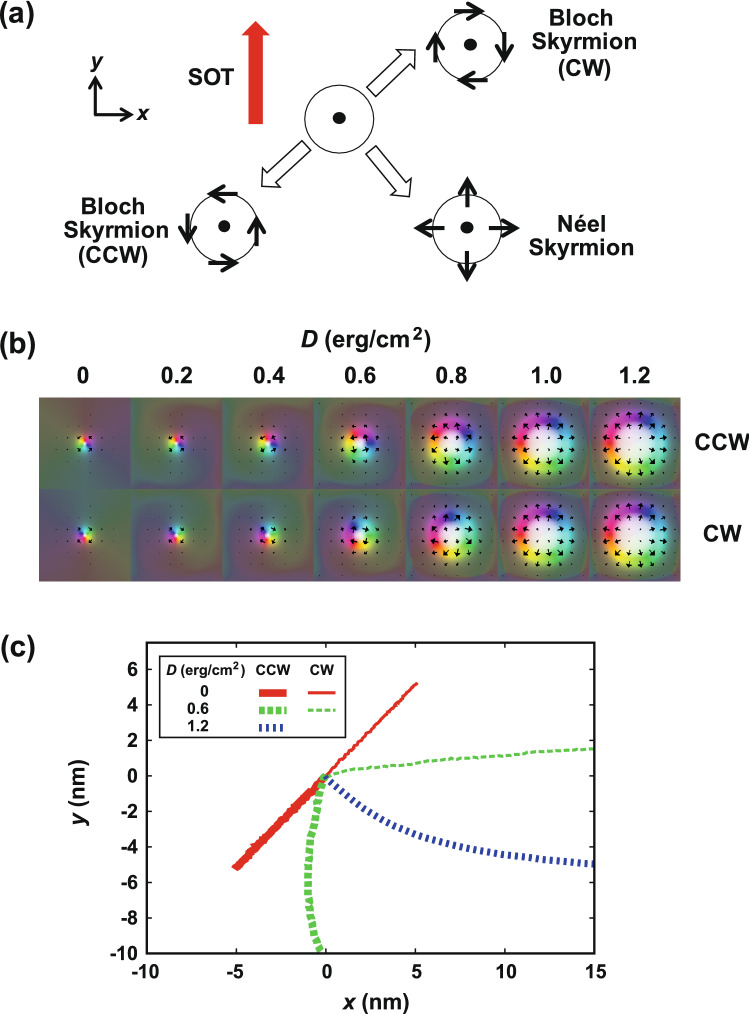
Motion of a skyrmion by SOT. (**a**) Direction of a motion of Bloch type skyrmions (CW and CCW) and Néel type skyrmion by SOT (analytical result^31^). (**b**) Change of the skyrmion structures for CCW and CW by the *D* value. (c) Change of the motion direction of a skyrmions for CCW and CW by the *D* value (simulation results).

First, we show the change of the direction of the motion of the Néel and Bloch type skyrmions by SOT depending on the chirality by analytical model^31^. Figure 1 shows representative results. In the case of the Bloch type skyrmion, the direction of motion by SOT changes 180º depending on the chirality [see Fig. 1a]. This means that we can differentiate the chirality of the Bloch type skyrmions by detecting the opposite directions of motion. On the other hand, the direction of motion of the Néel type skyrmion is 90º, which requires further consideration as detailed below.

In the magnetic thin film, not only the diameter of the skyrmion, but also the direction of the magnetization on the domain wall surrounding the skyrmion changes by the *D* value. As shown in Fig. 1b, a Bloch type skyrmion in which the magnetization is parallel to the domain wall appears for the *D* = 0 erg/cm^2^ case. The magnetization changes as the *D* value increases, and Néel type skyrmion appears for the *D* = 1.2 erg/cm^2^ case.

Figure 1c shows the simulated results of the change of the direction of the skyrmion motion by the *D* value and chirality. For the *D* = 0 erg/cm^2^ cases, the same results with the analytical model are obtained. However, the directional angle dependent upon the chirality decreases with increasing the *D* value. In our previous paper^21^, in which we reported the control of the chirality by a heat pulse, we use the Bloch type skyrmion with *D* = 0.6 erg/cm^2^. In that case, the clockwise (CW) skyrmion moves to the + *x* direction by SOT, however the counter clockwise (CCW) skyrmion moves to the –*y* direction predominantly (Fig. 1c, *D* = 0.6 erg/cm^2^). It shows that the branch structure to discriminate the chirality of the skyrmion should be designed considering the *D* value.

**Figure 2:**
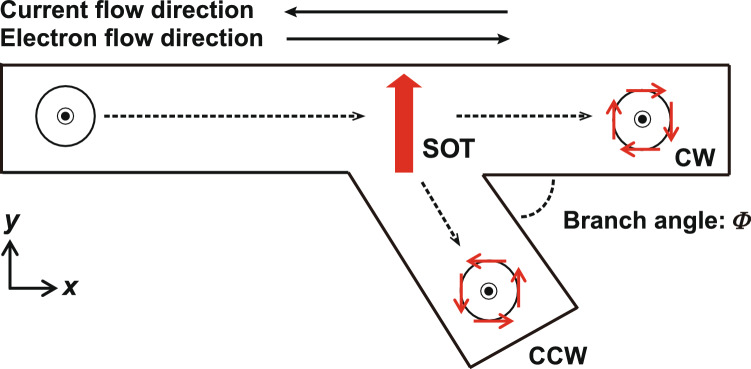
Illustration of a nanowire with a branch to discriminate a skyrmion chirality.

Figure 2 shows the branch structure to discriminate the chirality of the Bloch type skyrmion. In this figure, the current flows to the –*x* direction (electron moves to the + *x* direction), and the skyrmion moves to the + *x* direction by STT. When SOT with the + *y* direction is also applied, the CW skyrmion with *D* = 0.6 erg/cm^2^ moves to the + *x* direction as mentioned before. It moves to the + *x* direction at the branch, moving into the upper branch. On the other hand, the CCW skyrmion moves to the –*y* direction by SOT, moving to the below branch by the combination of STT and SOT.

**Figure 3:**
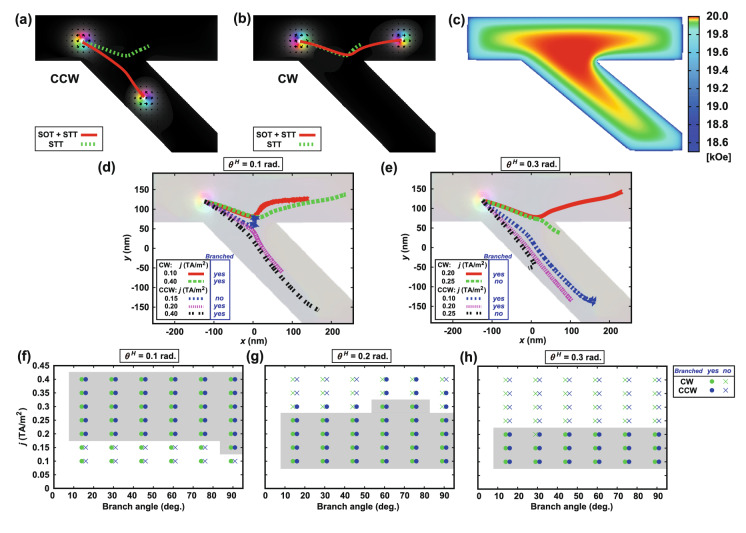
Motion of a skymion by SOT and STT. (**a**,**b**) Motion of a (**a**) CCW and (**b**) CW skymions by SOT and STT for 40 ns with the branch angle of 45º, *j* = 0.3 × 10^12^ A/m^2^, *P* = 0.7, ${\theta }^{H}=$ 0.1 rad (red lines). The green dashed lines show the cases without SOT (${\theta }^{H}=$ 0 rad). (**c**) Distribution of the demagnetizing field at the branch. (**d**,**e**) Change of the skyrmion motion by the current density and spin Hall angle ${\theta }^{H}=$ (**d**) 0.1 and (**e**) 0.3 rad. “Branched no” shows the cases for unbranch and a skyrmion crashed at the strip edge or branch center. (**f**–**h**) Phase diagrams of the branch. The branch angle was varied from 15 to 90º, and the current was varied from 0.1 to 0.4 TA/m^2^. The spin Hall angles are ${\theta }^{H}=$(**f**) 0.1, (**g**) 0.2 and (**h**) 0.3 rad. Circles show the branched cases, and the crosses show the unbranched cases. Green (blue) symbols represent the CW (CCW) skyrmion cases. Shaded area shows the angle and current ranges of the main branch to be achieved for the both types of skyrmions.

We then investigate the change of the branch directions by chiralities using simulation. Figure 3 shows the results. Figure 3a,b show the motion of the CCW and CW skyrmions for 40 ns, respectively. Here, the branch angle is 45º, and the current density is *j* = 0.3 × 10^12^ A/m^2^ with *P* = 0.7 and ${\theta }^{H}=$ 0.1 rad (red lines) or 0 rad (green dashed lines). These figures show that the direction of motion and the resulting branches for detection changes by the chirality and SOT (red line). The figures also show the simulated results without SOT (green dashed lines). The CW and CCW skyrmions move almost straight with minor departure by the branch. These results confirm that the branch direction does not change only by the chirality and SOT is required to control the branch. In all cases, the skyrmion moves to the center of the branches by the demagnetizing field and then moves along the branch. Figure 3c shows the demagnetizing field in the branch. The field becomes the maximum at the branch center because the width of the strip is maximum. That is the stable point for the skyrmion, and hence the skyrmion moves to the center of the branch first and then it moves along the branch.

Figure 3d,e show the change of the skyrmion motion by the current density and the spin Hall angle for 80 ns. The current was varied between 0.1 and 0.4 × 10^12^ A/m^2^ and the spin Hall angle of 0.1 [see Fig. 3d] and 0.3 rad [see Fig. 3e]. For the small current cases (*j* = 0.1 and 0.15 × 10^12^ A/m^2^), it takes long time to pass through the branch. As an example, the motions for 160 ns is shown in the figure. In Fig. 3d, the CW skyrmion moves to the upper branch for all cases. The CCW skyrmion moves to the bottom branch when the current is larger than 0.20 × 10^12^ A/m^2^, however it crashes the branch center and disappears when *j* = 0.15 × 10^12^ A/m^2^. In Fig. 3e, the CW skyrmion moves to the upper branch when the current is smaller than 0.2 × 10^12^ A/m^2^, however it moves to the lower branch and crashes into the strip edge and disappears when the current is larger than 0.25 × 10^12^ A/m^2^. The CCW skyrmion moves to the lower branch for all cases, however it crashes into the strip edge and disappear when the current is larger than 0.25 × 10^12^ A/m^2^. Note that SOT is required to change the motion direction. However, when the spin Hall angle is large (~ 0.3 rad), the current range for the main branch decreases by misdirectional motion or anihilation. These results show that the spin Hall angle of 0.1 rad is sufficient for the chirality discrimination, which is experimentally achievable^30,39^ and larger angle is not necessary.

Figure 3f–h show the phase diagram of the branch. Here, the branch angle and the current are varied between 15 and 90º, and between 0.1 and 0.4 × 10^12^ A/m^2^, respectively. The circles show the branched cases and the crosses show the unbranched cases. The green (blue) symbols show the CW (CCW) skyrmion cases. The shaded regions show the angle and current ranges for the CW and CCW skyrmions to branch. Note that 0.1 × 10^12^ A/m^2^ is the minimum value we used in the simulation. Therefore, it is not the true minimum for normal operation. These figures show that the effect of the branch angle on the current range is small. The current range decreases because the maximum current decreases as the spin Hall angle increases. The misdirectional motion of the skyrmion does not depend on the branch angle, which is the same as that in Fig. 3d,e.

From these results, the skyrmion chiralities are proven to be discriminated electrically by SOT and STT. The branch direction of the Bloch type skyrmion moving by SOT and STT changes depending on the chirality. The spin Hall angle of 0.1 rad is sufficient for the chirality discrimination, which is experimentally achievable and larger angle is not necessary. The effect of the branch angle on the current range is minor, which is advantageous for device miniaturization.

In general, the trajectories of the skyrmion are not parallel to the electron flow direction by the Magnus force. The angle between the trajectories and the electron flow is determined by the ratio between the Gilbert damping constant, α, and the parameter for STT, β^9^. When β = α, the corresponding angle become zero, and the skyrmion moves parallel to the electron flow direction. The angle also changes by the chirality with SOT. This study shows that the chirality of the skyrmion can be determined by using the change of the angle by chirality. Note that in this study, we selected β = α for simplicity. For β ≠ α case, we can also determine the chirality by adjusting the branch angle.

Here, the influence of the Joule heating induced by an electrical current flow can be ignored due to the following reason. In the branched system here, the current density becomes strongly inhomogeneous. It is expected that the temperature induced by the Joule heating by the distribution of the current density increases and influences the chirality. This can be similar to the case we previously reported where the chiralities of the skyrmion can be switched by a local pulse heat spot with realistic parameters, a Gaussian shape with a diameter of about 60 nm, the maximum temperature of 550 K and a pulse width of 10 ns^21^. However in the branch geometry with the width of 128 nm and the length of 512 nm for simulation, temperature may be dissipated within large area and the corresponding temperature gradient is much smaller than that with the heat spot we reported previously. It is hence concluded that the chirality does not change by the thermal effect in this study.

## Conclusions

We proposed a method to electrically discriminate the chirality of a skyrmion using a combination of STT and SOT. This proposed method was investigated and confirmed by micromagnetic simulation. This method is found to be highly effective to realize skyrmionics memory and logic devices with using the chirality of the skyrmion, allowing to achieve multiple-valued operation in combination with the presence and polarity of the skyrmion.

## Data Availability

The datasets generated during the current study are available from the corresponding author on reasonable request.
